# Gut Microbiota for Health: How Can Diet Maintain A Healthy Gut Microbiota?

**DOI:** 10.3390/nu12113596

**Published:** 2020-11-23

**Authors:** Cinzia Ferraris, Marina Elli, Anna Tagliabue

**Affiliations:** 1 Laboratory of Food Education and Sport Nutrition, Department of Public Health, Experimental and Forensic Medicine, University of Pavia, 27100 Pavia, Italy; 2 Human Nutrition and Eating Disorder Research Center, Department of Public Health, Experimental and Forensic Medicine, University of Pavia, 27100 Pavia, Italy; 3 AAT-Advanced Analytical Technologies Srl, 29017 Fiorenzuola d'Arda, Italy; marina.elli@aat-taa.eu

The human intestinal tract is colonized by a resilient integrated ecosystem represented by a complex consortium of trillions of microbes. Overall, five phyla dominate the bacterial composition of the gut microbiota with the 90% of phylotypes identified in Firmicutes and Bacteroidetes, whereas Actinobacteria, Proteobacteria, and Verrucomicrobia are minor components. Each microorganism within the gut encodes unique metabolic functions which taken together comprise the gut microbiome. Indeed, the metabolic potential of the gut metagenome, or the sum of microbial encoded genes is about 100-fold that of the human genome, and even with functional redundancy between different microbial species, represents a metabolic potential rivaling that of the liver both in chemical diversity and impact on host health [1].

The gut microbiota is a highly dynamic system with its density and composition affected by many exogenous (e.g., diet, drugs, infections, environmental factors) and endogenous (e.g., age, sex, host genetic features) influencing factors. All of the environmental factors may uniquely influence the microbiota composition, but it has been shown that diet, in particular, plays a major role in determining the composition and changes of the gut microbiota [2].

Specifically, compositional pattern of the gut microbiota has been associated with habitual diets. In 2014, David et al. [3] demonstrated that the short-term consumption of diets composed entirely of animal or plant products alters microbial community structure and overwhelms inter-individual differences in microbial gene expression. The animal-based diet increased the abundance of bile-tolerant microorganisms (*Alistipes, Bilophila, and Bacteroides*) and decreased the levels of Firmicutes that metabolize dietary plant polysaccharides producing butyrate (*Roseburia, Eubacterium rectale, and Ruminococcus bromii*). Western diets, which are mainly omnivore-type diets apparently lead to a composition of the microbiota that is more associated with different types of disease. On contrary, the Mediterranean diet (MD) that has a very large fiber content and bioavailability, particularly in terms of insoluble fiber, being more than twofold higher than in a typical Western diet, has been associated with a beneficial microbiome related metabolomics profiles [4]. 

Other specific changes have been observed in therapeutical dietary interventions such as low-fermentable, oligo-, di-, mono-saccharides and polyols (FODMAPs), ketogenic (KD), and gluten-free (GFD) diets prescribed in irritable bowel syndrome (IBS), celiac disease (CD), or neurological disorders (ND). These kinds of diets are characterized by a reduction or exclusion of a specific nutrient from the entire dietary pattern. Despite these alimentary regimens showing beneficial effects on disease symptoms, they can affect microbiota composition, especially if they are protracted for a long time [5].

However, due to the complexity of the food composition (macronutrients, micronutrients, bioactive compounds) and their interactions, it is very difficult to identify the effects of single dietary nutrients in the context of the different dietary patterns in habitual or therapeutical diets. 

In vitro models are used ad preclinical tools to assess the impact of specific food components on human gut mucosa by mimicking digestion, absorption and intestinal fermentation. Several in silico models could be interconnected and used in the future to predict the effect of food systems on various metabolic responses. The huge number of scientific reports about in vitro gastrointestinal digestion systems in the latest 40 years indicated the interest in understanding the behavior of food and food components during human digestion [6]. However, these models are in some way unable to reproduce overall in vivo digestion events; therefore, they have to be validated by in vivo assays, providing insights about critical parameters. 

A very interesting review article published by Yang in Nutrients [7] summarized the available evidence on the effects of the several dietary nutrients (carbohydrates protein and lipids, vitamins, minerals, and polyphenols) on three gut microbiota characteristics: shifts in potentially beneficial and potentially detrimental gut microbiota, the Firmicutes/Bacteroidetes ratio, and microbial diversity. The authors considered all the scientific studies published between 2005 and 2019 including in vitro and in vivo models and human clinical trials when available. In particular, studies reported by Yang et al. [7] confirm that carbohydrates form the major modulator for health beneficial microbes. For example, fermentable dietary fibers, which include AX, RS, inulin, oligosaccharides, and GOS, increase the abundance of *Bifidobacterium, Lactobacillus, Roseburia, Bacteroides, Akkermansia,* butyrate-producing *Fecalibacterium, and Ruminococcus* at the genus level, which are associated with various health benefits, whereas diets containing more than 44% energy from fat increase the ratio of F/B, which can be attenuated by red wine/tea-derived polyphenols. Major groups of polyphenols assayed in both in vitro and preclinical studies have shown their ability to modulate the gut microbiota to a beneficial pool characterized by the abundance of *Bifidobacterium, Lactobacillus, Akkermansia,* and *Fecalibacterium sp*. The beneficial mechanisms observed in those studies were mainly attributed to the production of SCFAs and other bacterial metabolites that contributed towards positive changes in gut health and reducing the inflammatory process, thereby improving systemic disease status. 

The authors synthesized the role of some micronutrients (vitamin D, calcium) in modulating gut microbiota toward a healthy phenotype but underline a scarcity in the literature that explores the role of minerals and trace elements in modulating gut microbiota. The use of over-the counter dietary supplements of vitamins and minerals is increasing among the public; it is therefore extremely useful to understand their potential interaction with the gut microbiota which is not always positive. 

Following the work of Yang et al. [7] a few interesting reviews focused on the interaction of diet/microbiome as the core mechanism by which gut microbes affect host brain function [8]. As described by the authors, micronutrients, and macronutrients have a pivotal role in neurodevelopment, brain function, and they are involved in modulating various behavioral phenotypes. The microbiome, in contrast, is both affected by the diet, utilizes nutrients as metabolic precursors, and also alters its nutritional content, as different bacteria can synthesize or utilize nutrients in the host diet. Nutrient-microbiome-host interactions therefore provide an overarching framework to understand the function of the gut-brain axis, as suggested by Ezra-Nevo G and colleagues [8] and previously reported by Iannone et al. [9] in neuropsychiatric disorders. 

The final question regarding the impact of the total diet on gut microbiota remains partly unanswered but we have many elements that will be useful to depict the picture. Diet, especially high intake of fermentable fibers and plant polyphenols, appears to regulate microbial activities within the gut, supporting regulatory guidelines encouraging increased consumption of whole-plant foods (fruit, vegetables, and whole-grain cereals), and providing the scientific rationale for the design of efficacious prebiotics. 

Due to complexity of multifactorial diet–microbiota studies, novel tools for extracting useful information from large data sets (machine learning) are required to predict specific food consumption pattern by measuring bacterial biomarkers, combined with fecal food metabolites and indicators of the immune and mucosal status of the gut [10]. This holistic approach appears to be very useful to identify new biomarkers and to understand the role of food in health and disease, as well as to identify inter-individual variations useful to formulate personalized dietary recommendations [11]. Fecal bacteria were recently studied as biomarkers of food intake for specific whole foods with high predictive accuracy by Shinn et al. [12].

Health care professionals should be educated on the important role of dietary choices in modulating gut microbiota for both preventive and therapeutically purposes. The maintenance of a healthy gut microbiota, via nutrition and use of food supplements, could lead to the reduction of the metabolic risk associated to dangerous lifestyles.

## References

[1] Thursby E., Juge N. (2017). Introduction to the Human Gut Microbiota. Biochem. J..

[2] Zmora N., Suez J., Elinav E. (2019). You Are What You Eat: Diet, Health and the Gut Microbiota. Nat. Rev. Gastroenterol. Hepatol..

[3] David L.A., Maurice C.F., Carmody R.N., Gootenberg D.B., Button J.E., Wolfe B.E., Ling A.V., Devlin A.S., Varma Y., Fischbach M.A. (2014). Diet Rapidly and Reproducibly Alters the Human Gut Microbiome. Nature.

[4] Tosti V., Bertozzi B., Fontana L. (2018). Health Benefits of the Mediterranean Diet: Metabolic and Molecular Mechanisms. J. Gerontol. Ser. A.

[5] Tagliabue A., Ferraris C., Uggeri F., Trentani C., Bertoli S., de Giorgis V., Veggiotti P., Elli M. (2017). Short-Term Impact of a Classical Ketogenic Diet on Gut Microbiota in GLUT1 Deficiency Syndrome: A 3-Month Prospective Observational Study. Clin. Nutr. ESPEN.

[6] Lucas-González R., Viuda-Martos M., Pérez-Alvarez J.A., Fernández-López J. (2018). In Vitro Digestion Models Suitable for Foods: Opportunities for New Fields of Application and Challenges. Food Res. Int..

[7] Yang Q., Liang Q., Balakrishnan B., Belobrajdic D.P., Feng Q.-J., Zhang W. (2020). Role of Dietary Nutrients in the Modulation of Gut Microbiota: A Narrative Review. Nutrients.

[8] Ezra-Nevo G., Henriques S.F., Ribeiro C. (2020). The Diet-Microbiome Tango: How Nutrients Lead the Gut Brain Axis. Curr. Opin. Neurobiol..

[9] Iannone L.F., Preda A., Blottière H.M., Clarke G., Albani D., Belcastro V., Carotenuto M., Cattaneo A., Citraro R., Ferraris C. (2019). Microbiota-Gut Brain Axis Involvement in Neuropsychiatric Disorders. Expert Rev. Neurother..

[10] Celi P., Verlhac V., Pérez Calvo E., Schmeisser J., Kluenter A.-M. (2019). Biomarkers of Gastrointestinal Functionality in Animal Nutrition and Health. Anim. Feed Sci. Technol..

[11] Picó C., Serra F., Rodríguez A.M., Keijer J., Palou A. (2019). Biomarkers of Nutrition and Health: New Tools for New Approaches. Nutrients.

[12] Shinn L.M., Li Y., Mansharamani A., Auvil L.S., Welge M.E., Bushell C., Khan N.A., Charron C.S., Novotny J.A., Baer D.J. (2020). Fecal Bacteria as Biomarkers for Predicting Food Intake in Healthy Adults. J. Nutr..

